# Combination of automated brain volumetry on MRI and quantitative tau deposition on THK-5351 PET to support diagnosis of Alzheimer’s disease

**DOI:** 10.1038/s41598-021-89797-x

**Published:** 2021-05-14

**Authors:** Minjae Kim, Sang Joon Kim, Ji Eun Park, Jessica Yun, Woo Hyun Shim, Jungsu S. Oh, Minyoung Oh, Jee Hoon Roh, Sang Won Seo, Seung Jun Oh, Jae Seung Kim

**Affiliations:** 1 Department of Radiology and Research Institute of Radiology, Asan Medical Center, University of Ulsan College of Medicine, 88 Olympic-ro 43-gil, Songpa-Gu, Seoul, 05505 South Korea; 2 Department of Nuclear Medicine, Asan Medical Center, University of Ulsan College of Medicine, 88 Olympic-ro 43-gil, Songpa-Gu, Seoul, 05505 South Korea; 3 Department of Neurology, Asan Medical Center, University of Ulsan College of Medicine, Seoul, 05505 South Korea; 4 Department of Neurology, Samsung Medical Center, Sungkyunkwan University School of Medicine, 81 Irwon-ro, Kangnam-ku, Seoul, 06351 South Korea

**Keywords:** Diagnostic markers, Alzheimer's disease

## Abstract

Imaging biomarkers support the diagnosis of Alzheimer’s disease (AD). We aimed to determine whether combining automated brain volumetry on MRI and quantitative measurement of tau deposition on [18F] THK-5351 PET can aid discrimination of AD spectrum. From a prospective database in an IRB-approved multicenter study (NCT02656498), 113 subjects (32 healthy control, 55 mild cognitive impairment, and 26 Alzheimer disease) with baseline structural MRI and [18F] THK-5351 PET were included. Cortical volumes were quantified from FDA-approved software for automated volumetric MRI analysis (NeuroQuant). Standardized uptake value ratio (SUVR) was calculated from tau PET images for 6 composite FreeSurfer-derived regions-of-interests approximating in vivo Braak stage (Braak ROIs). On volumetric MRI analysis, stepwise logistic regression analyses identified the cingulate isthmus and inferior parietal lobule as significant regions in discriminating AD from HC and MCI. The combined model incorporating automated volumes of selected brain regions on MRI (cingulate isthmus, inferior parietal lobule, hippocampus) and SUVRs of Braak ROIs on [18F] THK-5351 PET showed higher performance than SUVRs of Braak ROIs on [18F] THK-5351 PET in discriminating AD from HC (0.98 vs 0.88, *P* = 0.033) but not in discriminating AD from MCI (0.85 vs 0.79, *P* = 0.178). The combined model showed comparable performance to automated volumes of selected brain regions on MRI in discriminating AD from HC (0.98 vs 0.94, *P* = 0.094) and MCI (0.85 vs 0.78; *P* = 0.065).

## Introduction

Imaging biomarkers play an important role in supporting the diagnosis of Alzheimer’s disease (AD), not only in the research field but also in clinical practice. With the advent of amyloid and tau PET ligands, there has been a huge advance in the understanding of pathophysiologic mechanism of AD, and early diagnosis of AD can be made even in the preclinical or prodromal stage^1^. Amyloid and tau PET enable in vivo visualization of the underlying pathophysiologic culprits of AD, namely senile plaques and neurofibrillary tangles, but their clinical use may be affected by its availability, cost and consideration of ionizing radiation exposure. MRI is used for standard practice to support the diagnosis of AD and to exclude other causes of cognitive impairment such as stroke, vascular dementia, normal pressure hydrocephalus, or inflammatory or neoplastic conditions. Atrophy of the medial temporal lobe, particularly the hippocampus and entorhinal cortex, was shown to be important in patients on the AD spectrum and these regions were identified as predictors of time to progression from mild cognitive impairment (MCI) to AD^2–6^. Qualitative visual assessment of atrophy on MRI suffers from poor interobserver agreement, which is a major limitation for implementing it in a clinical setting^7,8^. This limitation can be addressed by quantitative volumetry analysis, and automated software programs for quantitative volumetric analysis have been developed^9,10^. Extensive validation of these software packages has been done for clinical use with comparison to manually segmented volumetric measurements^11–14^, and commercially available automated brain volumetric tool showed high correlation in brain volume measurement with tools extensively used in research settings with moderate to high sensitivity (63.3–83%) and high specificity (93–100%) in differentiating AD from healthy controls (HC)^15–18^.

[18F] THK-5351 is one of the first generation of tau PET tracers^19^ and a close correspondence between the Braak staging of tau pathology and retention of the first generation tau PET tracers has been demonstrated in AD^20,21^. Braak stages of tau pathology, derived from cross-sectional data, proposed how AD-related tau pathology begin in medial temporal structures extending to limbic areas, posterior cingulate cortex and then widely to isocortical brain areas^22–24^. While tracer specificity related to off-target binding of [18F] THK5351 to monoamine oxidase B (MAO-B) remains as a possible limitation, previous studies reported that binding of [18F] THK5351 in the hippocampus was not influenced by off-target binding in the choroid plexus unlike [18F] AV-1451^19,25–29^. Moreover, longitudinal analysis of tau PET demonstrated that measurement of changes in the tau PET SUVR can be used as an efficient outcome measure in disease modifying clinical trials^30,31^.

While tau PET reflects the underlying pathophysiologic hallmark of AD, MRI demonstrates regional atrophy with automated brain volumetry extensively validated for clinical use in AD. To our knowledge, no direct comparison has been performed between commercially available automated brain volumetry on MRI and [18F] THK-5351 PET in discrimination of AD spectrum. The purpose of this study was to compare commercially available automated brain volumetry on MRI and [18F] THK-5351 PET, and determine if the combination of these tools can aid discrimination of AD spectrum in a clinically feasible setting.

## Methods

All the experimental protocols were approved by institutional review boards of Asan Medical Center and Samsung Medical Center, Seoul, South Korea. All relevant study protocols for involving humans were in accordance with guidelines of institutional ethics committees.

### Participants

**Figure 1:**
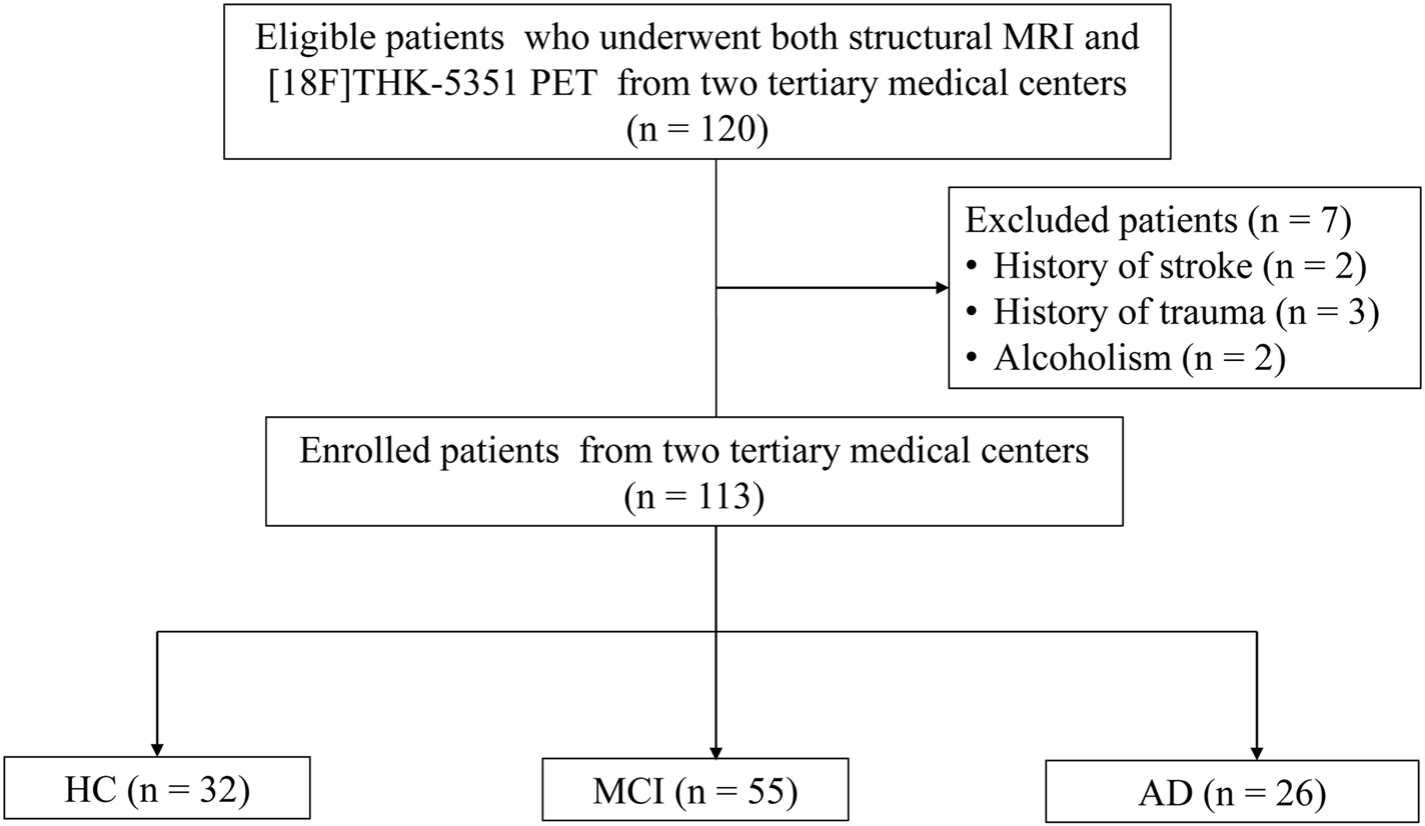
Flow diagram of the study population.

From a prospective cohort of a multicenter clinical trial (NCT02656498), 113 participants who had both baseline structural MRI and [18F] THK-5351 PET were included. All participants or their appropriate representatives provided informed consent, and all participants were examined under the protocols approved by the institutional review board of the two tertiary medical centers (Asan Medical Center and Samsung Medical Center). A flow diagram of participant inclusion is shown in Fig. 1. Participants with MCI and AD underwent structural MRI and [18F] THK-5351 PET at the time of initial diagnosis and the maximum time interval between the structural MRI and [18F] THK-5351 PET was 7 months. All participants underwent standard dementia screening, including recording of medical history, physical examination, brain MRI, [18F] THK-5351 PET, and neuropsychological testing. Clinical diagnosis was made according to standard research criteria for probable AD^32^ and MCI^33^, applied across a multidisciplinary team. Participants assigned as HC had a Korean version of Mini-Mental State Examination (K-MMSE) score > 1.5 standard deviation of the normative means^34,35^ and without current clinical depression (Geriatric Depression Scale < 11). Participants were excluded if they had a history of stroke, head trauma, or alcoholism. The 113 finally enrolled participants included 32 HC, 55 participants with MCI, and 26 participants with AD. The demographics of the study participants are provided in Table 1.

**Table 1:** Demographics of the study participants.

	HC	MCI	AD	*P* value
N	32	55	26	
Age (years)	69.4 ± 6.0	70.8 ± 7.1	63.6 ± 10.6	0.001
Sex (M/F)	11/21	20/35	11/15	0.816
Education (years)	10.6 ± 4.9	11.1 ± 4.6	10.6 ± 4.7	0.846
APOE ε4 (0/1/2)^a^	(24/7/0) (NA 1)	(28/17/5) (NA 5)	(14/6/2) (NA 4)	0.082
K-MMSE score	28.6 ± 1.2	25.0 ± 3.2	19.9 + 5.0	< 0.001
Days between MRI and [18F] THK-5351 scan	47.5 ± 35.6	57.1 ± 50.1	53.6 ± 47.0	0.659

HC, healthy controls; MCI, mild cognitive impairment; AD, Alzheimer disease; K-MMSE, Korean version of the Mini-Mental State Examination.

^a^0/1/2 indicates non-carriers/heterozygotes/homozygotes for APOE ε4 respectively.

### MRI acquisition

A 3.0-T system MRI (Achieva; Philips Medical Systems, Best, The Netherlands) with an eight-channel sensitivity-encoding head coil was used. A high-resolution anatomical three-dimensional (3D) volume image was obtained using a 3D gradient-echo T1-weighted sequence. The following parameters were used: repetition time/echo time, 9.9/4.6 ms; flip angle, 8°; field of view, 224 × 224 mm; matrix, 224 × 224; and slice thickness, 1 mm with no gap^36^.

### [18F] THK-5351 PET imaging acquisition

All PET images were acquired using Discovery 690, 710, and 690 Elite PET/CT scanners (GE Healthcare; Milwaukee, WI, USA) at Asan Medical Center and a Discovery STE PET/CT scanner (GE Healthcare) at Samsung Medical Center. The identical imaging and reconstruction protocol was used in both centers. [18F] THK-5351 PET images were obtained for 20 min, starting 50 min after injection of 185 ± 18.5 MBq of [18F] THK-5351, which binds to the aggregated tau in paired helical filaments^36,37^. Prior to the PET scan, a head holder was applied to minimize head motion and brain CT images were obtained for attenuation correction. Using the ordered-subsets expectation maximization algorithm (iteration = 4, subset = 16), 3D PET images were reconstructed with a voxel size of 2.0 × 2.0 × 3.27 mm^3^. To increase data uniformity between different PET scanners, a 3D Hoffman phantom-based PET harmonization method was applied based on the results of correction factors obtained from phantom studies using 3D Hoffman brain phantom^38,39^.

### Quantitative image analysis

#### MRI volumetry

Automated MRI-volumetry analyses using NeuroQuant software package (CorTechs Labs, La Jolla, CA, USA) were performed via the standard processing pipeline. The details of this procedure are previously described elsewhere^11,40^. DICOM files were uploaded to the servers for processing. Briefly, the protocol entails a quality check, adjustment for gradient non-linearity/B1 field inhomogeneity, and skull stripping followed by a discrete cosine transformation and registration onto a dynamic probabilistic atlas (https://www.cortechslabs.com/whitepapers/). An anatomic label is designated to each voxel based on approximations from the dynamic probabilistic atlas that is structure-wise similar to the FreeSurfer but uses an independent code-based method for intensity normalization and gradient distortion correction to accommodate scanner-specific acquisition-level differences and to better represent the aged population. The MRI analysis provides a report that includes raw and corrected volumes (% intracranial volume) for 66 brain regions. All reports were reviewed to ensure adequate quality.

#### Tau PET

[18F] THK-5351 PET images were segmented by using the default automated gyral-based parcellation method of FreeSurfer (version 5.3.0; http://surfer.nmr.mgh.harvard.edu)^13,24^. The SUVR of the cerebral cortex was calculated using the SUV of the cerebellar cortex as a reference region. Bilateral Braak stage region of interest (ROI) were created by combining FreeSurfer ROIs into non-overlapping Braak regions, using the Desikan-Killiany cortical atlas^41^. Further analyses from the automatic FreeSurfer segmentation were utilized for the left and right hippocampus. The values for the left and right hemispheres were then averaged.

The ROIs of the in vivo Braak staging were expressed as Braak I/II, Braak III/IV and Braak V/VI, reflecting the propagation of tau pathology, which begins in the medial temporal structures, extends to limbic areas and then widely spreads into isocortical brain areas^22–24^. The mean SUVR of ROIs belonging to Braak I/II, Braak III/IV and Braak V/VI were calculated and used for the analysis. Braak I/II include entorhinal cortex and hippocampus. Braak III/IV include parahippocampal, fusiform, lingual, middle temporal, caudal anterior cingulate, rostral anterior cingulate, posterior cingulate, cingulate isthmus, insula, inferior temporal and temporal pole. Braak V/VI include superior frontal, lateral orbitofrontal, medial orbitofrontal, frontal pole, caudal middle frontal, rostral middle frontal, pars opercularis, pars orbitalis, pars triangularis, lateral occipital, supramarginal, inferior parietal, superior parietal, precuneus, superior temporal, transverse temporal, pericalcarine, postcentral, cuneus, precentral and paracentral. We excluded basal ganglia and thalamus from the Braak ROIs as these regions are likely to reflect extensive off-target binding^42^.

### Statistical analysis

#### Comparison of demographics and quantitative values

The clinical characteristics of the participants in each group (HC, MCI, and AD) were compared. Chi-square tests were used for categorical variables and analysis of variance (ANOVA) was used for continuous variables with post hoc pairwise comparisons where relevant.

The cortical volumes and SUVRs of Braak ROIs were subsequently compared between groups using ANOVA, and baseline age was used as a covariate. Post-hoc analysis between groups was also conducted. In order to account for multiple comparisons across the multiple cortical regions, false discovery rate (FDR)-corrected *P*-value < 0.05 was used. *P* values for SUVRs were Bonferroni-corrected. Normality of all ROIs and Braak staging was verified using Kolmogorov-Smirnova and Shapiro–Wilk tests.

##### Construction of a diagnostic model

In order to identify significant brain regions in differentiating AD from HC and MCI, univariate and multivariate logistic regression analysis were performed using a general linear model with baseline age as a covariate. Stepwise logistic regression analyses were performed, and regions identified as significant regions upon univariate analysis underwent multivariate fitting to determine the best combination of regions for discriminating AD from HC and MCI. Variance inflation factor was considered to check for multicollinearity of the variables included in the regression equations and variables with variance inflation factor greater than 5 were excluded. The volume of the hippocampus was included as selected brain regions as the hippocampus is a well-established brain region that is known to be affected in AD and its atrophy is one of the most commonly used imaging criteria used for diagnosing AD^2,6,43^. The combined model was constructed using volumes of selected brain regions and SUVRs of Braak ROIs.

##### Performance measurement

Performance of discriminating AD from HC and MCI was quantified by area under the receiver operating characteristics (AUROC). The performances of volumes of selected brain regions, SUVRs of Braak ROIs and a combined model were calculated. The performance of the hippocampus alone was also evaluated.

##### Additional analysis

In order to provide the same ROI categorization for automated volumes on MRI and SUVRs on [18F] THK-5351 PET, automated volumes on MRI were grouped into Braak I/II, Braak III/IV and Braak V/VI as previously described, and univariate and multivariate logistic regression analysis on SUVRs from [18F] THK-5351 PET was performed as previously described. Performance of automated volumes on MRI grouped into Braak ROIs and SUVR of selected brain region was also evaluated.

**Figure 2:**
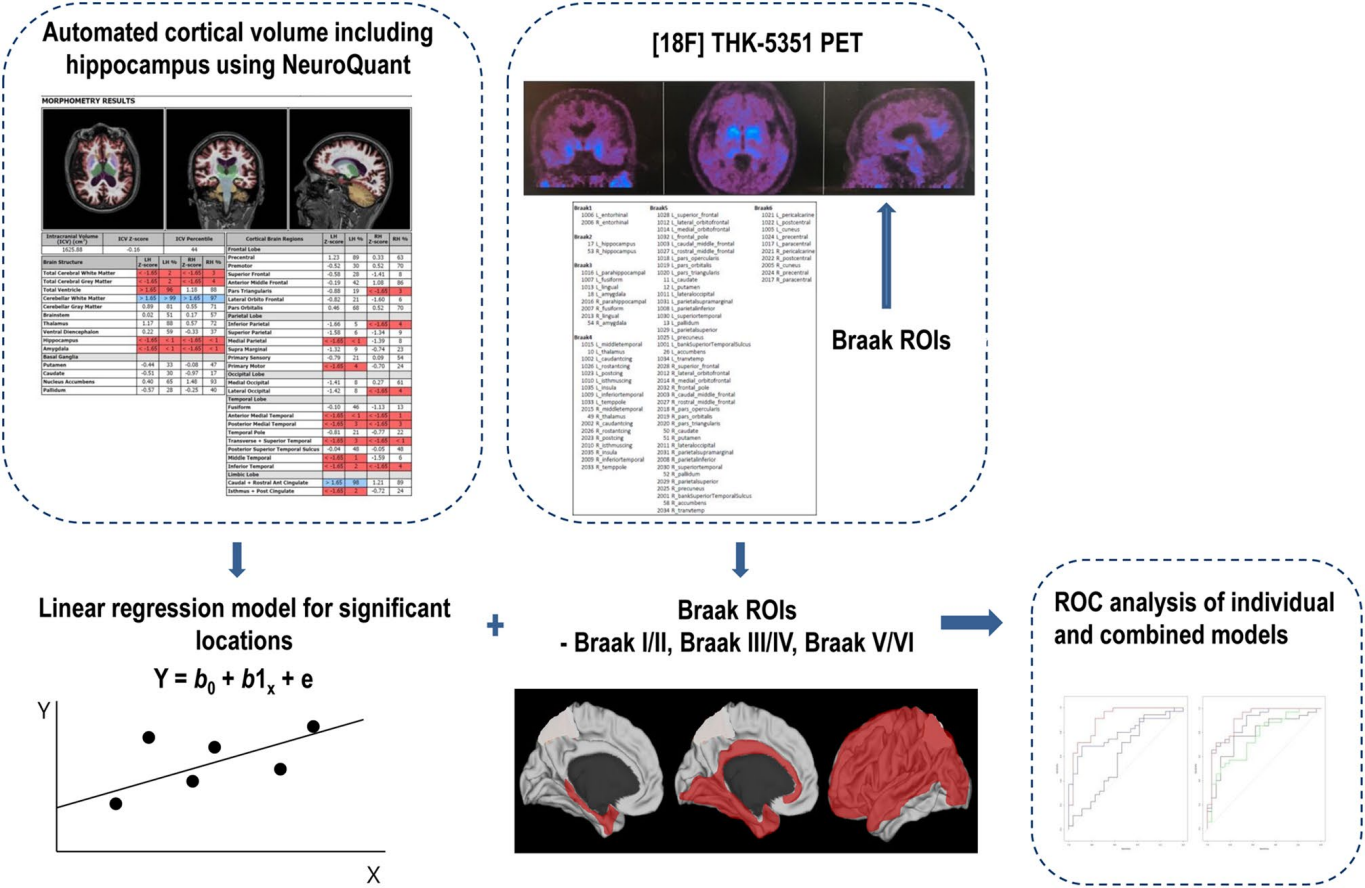
Overall process for image and statistical analysis. Automated MRI-volumetry analyses using NeuroQuant software package (CorTechs Labs, La Jolla, CA, USA, version 2.3, https://www.cortechslabs.com/products/neuroquant/) were performed via the standard processing pipeline. The ROIs of [18F] THK-5351 PET images were segmented and expressed as Braak I/II, Braak III/IV and Braak V/VI. The performances of individual and combined models were calculated. ROI = region of interest, ROC = receiver operating characteristics.

Statistical analyses were performed using R statistical software (version 3.3.3, R Core Team, Vienna, Austria). Figure 2 shows the overall process for image and statistical analysis.

## Results

### Participants

The demographics of the study participants are provided in Table 1. Of note, the mean age in the AD group was lower than in the HC and MCI groups (*P* = 0.001). The sex ratio, education level, and APOE allele variations were comparable across the three groups. In particular, there was no difference in the proportions of participants who are either homozygotes or heterozygotes for APOE allele variations between the AD and MCI groups [31% (8/26) vs 40% (22/55); *P* = 0.437]. As expected, the K-MMSE score was lower in the AD group than in the other groups (*P* < 0.001). The mean interval between [18F] THK-5351 PET and MRI was 53.6 days (range, 0–202 days; standard deviation, 47 days) with no significant difference among the three groups.

### Comparison of automated cortical volumes and SUVRs of Braak ROIs in AD vs. HC and AD vs. MCI

Automated volumes on MRI were compared and post-hoc analysis was performed between groups (Supplementary Table 1). Volumes of the following brain regions were different amongst the AD, MCI and HC groups on the ANOVA analysis: hippocampus (*P* = 0.015), entorhinal cortex (*P* = 0.001), superior temporal (*P* = 0.008), middle temporal (*P* = 0.001), basal ganglia (*P* = 0.011), amygdala (*P* = 0.011), anterior cingulate (*P* = 0.002), posterior cingulate (*P* =  < 0.001), cingulate isthmus (*P* =  < 0.001), anterior middle frontal (*P* = 0.004), inferior parietal lobule (*P* =  < 0.001) and supramarginal (*P* =  < 0.001). Upon post-hoc analysis, the AD group showed lower volume compared to the HC group in the hippocampus (*P* =  < 0.001), entorhinal cortex (*P* = 0.001), superior temporal (*P* =  < 0.001), middle temporal (*P* =  < 0.001), basal ganglia (*P* =  < 0.001), amygdala, *P* =  < 0.001), anterior cingulate (*P* = 0.006), posterior cingulate (*P* = 0.041), cingulate isthmus (*P* = 0.001), anterior middle frontal (*P* =  < 0.001), inferior parietal lobule (*P* =  < 0.001) and supramarginal (*P* =  < 0.001). Compared to the MCI group, the AD group showed lower volume in the hippocampus (*P* = 0.037), entorhinal cortex (*P* = 0.001), superior temporal (*P* = 0.001), middle temporal (*P* =  < 0.001), basal ganglia (*P* = 0.006), amygdala (*P* = 0.003), anterior cingulate (*P* = 0.013), posterior cingulate (*P* = 0.011), cingulate isthmus (*P* = 0.006), anterior middle frontal (*P* = 0.007), inferior parietal lobule (*P* = 0.005) and supramarginal (*P* = 0.021).

SUVRs of Braak ROIs were also compared and post-hoc analysis was performed between groups (Supplementary Table 1). The SUVRs were different amongst the HC, MCI and AD groups in Braak I/II (*P* = 0.011), Braak III/IV (*P* < 0.001) and Braak V/VI (*P* < 0.001). SUVRs of Braak V/VI was different in both AD vs. HC (*P* =  < 0.001) and AD vs. MCI (*P* = 0.002). SUVRs of Braak III/IV was different in AD vs. HC (*P* =  < 0.001) but not in AD vs. MCI (*P* = 0.231). SUVRs of Braak I/II was different in MCI vs. HC (*P* = 0.031).

### Significant brain regions among automated volumes on MRI for discriminating AD from HC and MCI

For discriminating AD from HC, the univariate analysis (Table 2) identified 9 brain regions (hippocampus, *P* =  < 0.001; entorhinal cortex, *P* = 0.012; middle temporal, *P* = 0.002; anterior cingulate, *P* = 0.013; posterior cingulate, *P* = 0.006; cingulate isthmus, *P* =  < 0.001; anterior middle frontal, *P* = 0.011; inferior parietal lobule, *P* < 0.001; supramarginal, *P* =  < 0.001) as significant regions. Upon the multivariate analysis (Table 2), the cingulate isthmus (*P* = 0.009) and inferior parietal lobule (*P* = 0.022) were identified as significant regions for discriminating AD from HC.

**Table 2:** Univariate and multivariate logistic regression analysis of automated volumes on MRI for discriminating AD from HC and MCI.

Variable	AD vs. HC				AD vs. MCI			
	Univariate analysis		Multivariate analysis		Univariate analysis		Multivariate analysis	
	β coefficient (95% CI)	*P* value	β coefficient (95% CI)	*P* value	β coefficient (95% CI)	*P* value	β coefficient (95% CI)	*P* value
Hippocampus	− 1.07 (− 1.76, − 0.51)	** < 0.001**			− 0.06 (− 0.46, 0.33)	0.782		
Entorhinal cortex	− 0.79 (− 1.46, − 0.25)	**0.012**			− 0.30 (− 0.74, 0.11)	0.172		
Superior temporal	− 0.14 (− 0.31, − 0.001)	0.061			− 0.02 (− 0.13, 0.08)	0.681	0.15 (0.0009, 0.32)	0.062
Middle temporal	− 0.34 (− 0.58, − 0.15)	**0.002**			− 0.12 (− 0.25, 0.005)	0.072		
Anterior cingulate	− 0.89 (− 1.62, − 0.26)	**0.013**			− 0.89 (− 1.57, − 0.28)	**0.006**	− 0.70 (− 1.50, 0.04)	0.072
Posterior cingulate	− 1.39 (− 2.51, − 0.48)	**0.006**			− 1.49 (− 2.51, − 0.63)	**0.001**		
Cingulate isthmus	− 1.88 (− 3.13, − 0.92)	** < 0.001**	− 2.64 (− 5.10, − 1.01)	**0.009**	− 1.22 (− 2.04, − 0.50)	**0.002**	− 0.94 (− 1.90, − 0.05)	**0.041**
Anterior middle frontal	− 0.38 (− 0.72, − 0.10)	**0.011**			− 0.18 (− 0.45, 0.05)	0.152		
Inferior parietal lobule	− 0.52 (− 0.84, − 0.28)	** < 0.001**	− 0.53 (− 1.07, − 0.15)	**0.022**	− 0.31 (− 0.50, − 0.14)	**0.001**	− 0.27 (− 0.50, − 0.07)	**0.012**
Supramarginal	− 0.79 (− 1.27, − 0.43)	** < 0.001**	− 0.56 (− 1.25, − 0.04)	0.061	− 0.18 (− 0.40, 0.01)	0.081		

For discriminating AD from MCI, the univariate analysis (Table 2) identified 4 brain regions (anterior cingulate, *P* = 0.006; posterior cingulate, *P* = 0.001; cingulate isthmus, *P* = 0.002; inferior parietal lobule, *P* = 0.001) as significant regions. Upon the multivariate analysis (Table 2), the cingulate isthmus (*P* = 0.041) and inferior parietal lobule (*P* = 0.012) were identified as significant regions for discriminating AD from MCI.

### Combined and individual performance of volumes of selected brain regions, SUVRs of Braak ROIs and volume of the hippocampus

Table 3 compares the combined and individual performance of volumes of selected brain regions (cingulate isthmus, inferior parietal lobule, hippocampus), SUVRs of Braak ROIs and volume of the hippocampus in discriminating AD from HC and MCI. In discriminating AD from HC, the combined model incorporating volumes of selected brain regions and SUVRs of Braak ROIs (AUROC 0.98, 0.95–1.00) showed comparable performance to volumes of selected brain regions (AUROC 0.94, 0.88–1.00; *P* = 0.094) but higher than SUVRs of Braak ROIs (AUROC 0.88, 0.79–0.98; *P* = 0.033) and volume of the hippocampus (AUROC 0.78, 0.65–0.91; *P* = 0.003).

**Table 3:** Comparison of combined and individual performance using volumes of selected brain regions, SUVRs of Braak ROIs and volume of the hippocampus in discriminating AD from HC and MCI.

	AUROC	P*	Sensitivity (%)	Specificity (%)	Accuracy (%)
**AD vs. HC**					
Combined model (volumes of selected brain regions + SUVRs of Braak ROIs)	0.98 (0.95, 1.00)		100	90.6	94.8
Volumes of selected brain regions (cingulate isthmus, inferior parietal lobe, hippocampus)	0.94 (0.88, 1.00)	0.094	88.5	90.6	89.7
SUVRs of Braak ROIs (Braak I/II, III/IV, V/VI)	0.88 (0.79, 0.98)	**0.033**	84.6	90.6	87.9
Volume of the hippocampus	0.78 (0.65, 0.91)	**0.003**	53.8	96.8	77.6
**AD vs. MCI**					
Combined model (volumes of selected brain regions + SUVRs of Braak ROIs)	0.85 (0.75, 0.94)		84.6	74.5	77.8
Volumes of selected brain regions (cingulate isthmus, inferior parietal lobe, hippocampus)	0.78 (0.68, 0.89)	0.065	75.4	80.0	75.3
SUVRs of Braak ROIs (Braak I/II, III/IV, V/VI)	0.79 (0.62, 0.92)	0.178	76.9	80.0	79.0
Volume of the hippocampus	0.48 (0.34, 0.63)	** < 0.001**	38.4	69.1	59.2

Numbers in parentheses are 95% confidence intervals. AUROC = area under the receiver operating characteristics curve.

*P* < 0.05 values are indicated in bold

*P-value refers to the significance between the differences of the AUROCs between the combined model and other model.

**Figure 3:**
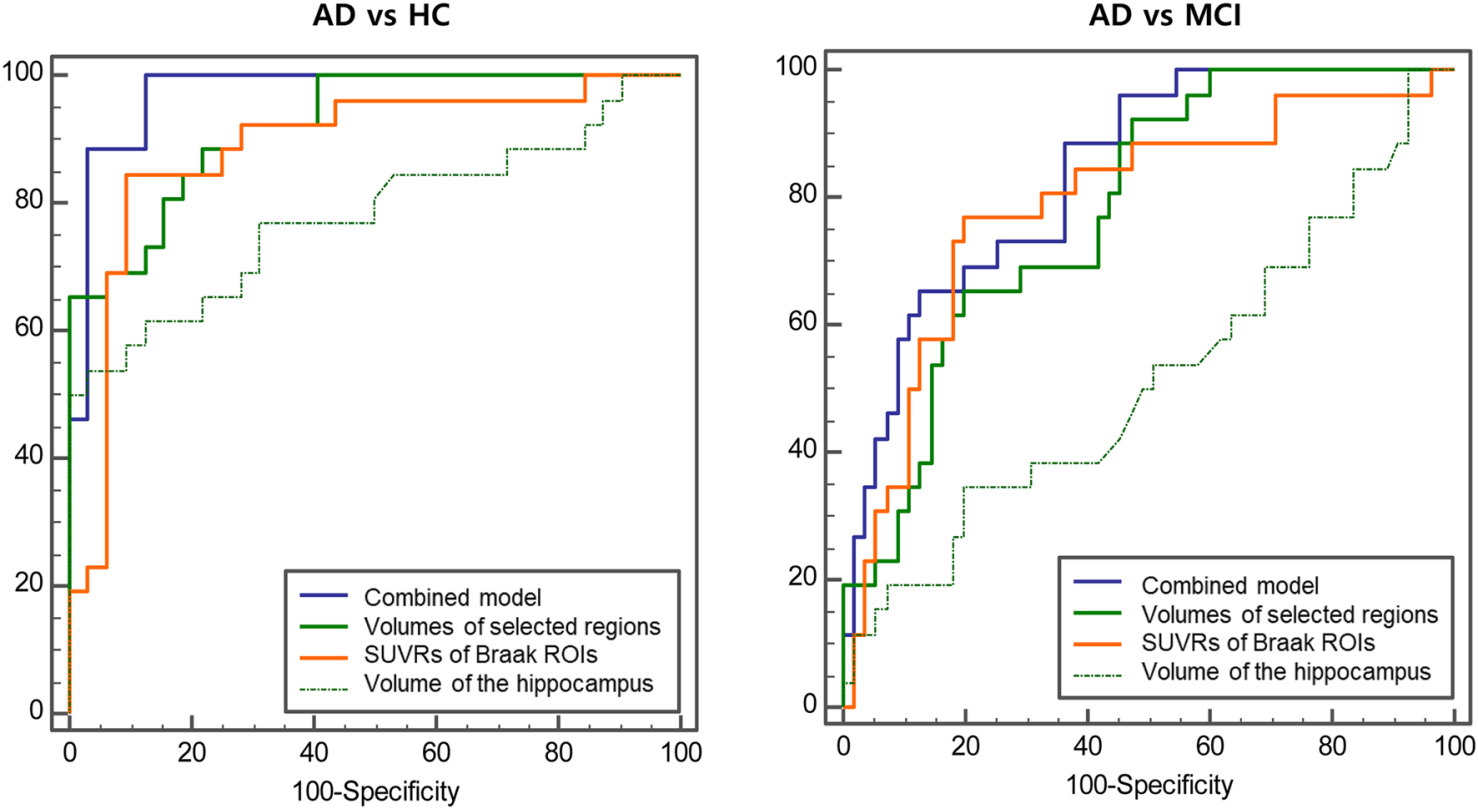
Combined and individual performance of volumes of selected brain regions, SUVRs of Braak ROIs and volume of the hippocampus in discriminating AD from HC and MCI. The performance was calculated using area under the receiver operating characteristics analysis. The performances of the combined model, volumes of selected brain regions (cingulate isthmus, inferior parietal lobule, hippocampus), SUVRs of Braak ROIs (Braak I/II, III/IV, V/VI) and volume of the hippocampus were compared in discriminating AD from HC (left) and MCI (right). AD = Alzheimer’s disease, HC = healthy control, MCI = mild cognitive impairment, ROI = region of interest.

In discriminating AD from MCI, the combined model incorporating volumes of selected brain regions and SUVRs of Braak ROIs (AUROC 0.85, 0.75–0.94) showed comparable performance to volumes of selected brain regions (AUROC 0.78, 0.68–0.89; *P* = 0.065) and SUVRs of Braak ROIs (AUROC 0.79, 0.62–0.92; *P* = 0.178). The combined model showed higher performance than volume of the hippocampus (AUROC 0.48, 0.34–0.63; *P* < 0.001). Figure 3 shows AUROC of the combined model and individual parameters in discriminating AD from HC and MCI.

### Additional analysis

The results of univariate and multivariate analysis of SUVRs from [18F] THK-5351 PET are provided in Supplementary Table 2. The supramarginal gyrus was identified as the significant region for discriminating AD from HC and MCI. The performance of SUVR of selected brain region and automated volumes on MRI grouped into Braak ROIs is shown in Supplementary Table 3.

## Discussion

In this study, performances of automated brain volumetry on MRI and quantitative measurement of tau deposition on [18F] THK-5351 PET were evaluated in discriminating AD on the AD spectrum. The combined model incorporating automated volumes of selected brain regions on MRI (cingulate isthmus, inferior parietal lobule, hippocampus) and SUVRs of Braak ROIs on [18F] THK-5351 PET showed higher performance than SUVRs of Braak ROIs on [18F] THK-5351 PET in discriminating AD from HC (0.98 vs 0.88, *P* = 0.033) but not in discriminating AD from MCI (0.85 vs 0.79, *P* = 0.178). The combined model showed comparable performance to automated volumes of selected brain regions on MRI in discriminating AD from HC (0.98 vs 0.94, *P* = 0.094) and MCI (0.85 vs 0.78; *P* = 0.065). The combined model showed higher performance than volume of the hippocampus in discriminating AD from HC (0.98 vs. 0.78; *P* = 0.003) and MCI (0.85 vs 0.48; *P* < 0.001). This underscores the robust performance of automated brain volumetry on MRI and quantitative measurement of tau deposition on [18F] THK-5351 PET in discrimination of AD spectrum with added value of automated brain volumetry in discriminating AD from HC.

While tau PET reflects the underlying pathophysiologic mechanism of AD, MRI demonstrates regional atrophy for which automatic volumetric assessment tool is available. In our study, commercially available, FDA-approved automated brain volumetry tool was evaluated that is being increasingly used in clinical settings as it is easily accessible providing fully automated service in a clinically acceptable time-period and feasible setting. Diagnostic efficacy of the automated volumetric assessment tool has previously been demonstrated in patients on the AD spectrum with high sensitivity (63.3–83%) and high specificity (93–100%) in differentiating AD from HC^5,6^, which is comparable to the performance demonstrated in our study (AUROC, 0.94; sensitivity, 88.5%; specificity, 90.6%). Previous studies used volume of the medial temporal lobe in the discrimination of AD while our study identified volumes of the cingulate isthmus and inferior parietal lobule by performing stepwise logistic regression analyses. The value of automated volumetric measurement of both the cingulate isthmus and inferior parietal lobule has been previously reported^6,16^, and the combination of automated volumetry of the entorhinal cortex and inferior parietal lobule was the best predictor of time to progress from MCI to AD^6^. Preferential atrophy of specific laminae in the inferior parietal lobule has been demonstrated in the early stages of AD on pathologic studies^44,45^. In addition, projections from the inferior parietal lobule include subfields within the medial temporal lobe reflecting spread of AD pathology from the temporal lobe to an interconnected region in the parietal lobe^46^.

While automated brain volumetry on MRI may be easily accessible in daily clinical practice, most studies have been performed in a research setting for tau PET. Currently there is no consensus for quantifying tau deposition on tau PET, and multiple tau-PET quantification methods (in vivo Braak staging, regional uptake in Braak composite regions, several whole-brain measures of tracer uptake, regional uptake in AD-vulnerable voxels and uptake in a priori defined regions) showed that all methods were related to amyloid and global cognition but regional measures covering AD-vulnerable regions increased sensitivity to early tau PET signal, atrophy and memory decline^47^. We based our analysis of [18F] THK-5351 PET according to the methods described by Scholl et al.^24^ who used weighted bilateral composite FreeSurfer-derived regions of interests approximating the anatomical definitions of transentorhinal (Braak stage I/II), limbic (III/IV) and isocortical (V/VI) Braak stages^24^. This reflects the propagation of tau accumulation through the course of the disease as supported by neuropathological data by Braak and Braak^22^. Off-target binding remains as an important issue with [18F] THK-5351 in relation to MAO-B with greatest reductions seen in SUVs in the basal ganglia and thalamus when MAO-B inhibitors were used^42^. While the heterogeneous MAO-B availability across the cortex may limit the interpretation of [18F] THK-5351, studies have demonstrated that no statistically significant reductions were seen using standard reference region-based approach^29^. In addition, significant tau tracer retention in the temporal lobe as well as extra-temporal regions has been reported even in cognitively normal older population supporting the primary age-dependent tauopathy despite some controversy regarding this entity^24,48,49^.

There were several limitations in our study. First, there were relatively small number of patients with AD (23%, 26/113) and overrepresentation of MCI (49%, 55/113). Second, age correction was not performed in the quantitative analysis of Braak ROIs from [18F] THK-5351 PET. It has been shown that tau PET SUVR demonstrated modest association with age throughout most regions of the brain in HC^48^ but currently there is no consensus or established methods and reference standards for age correction in the quantitative analysis of Braak ROIs. Most relevant studies relate to [18F] AV-1451 and further work is required pertaining this issue in relation to [18F] THK-5351. Third, the mean age in the AD group was significantly lower than in the HC and MCI group suggesting the inclusion of early onset AD patients in the study. However, despite alleged difference in the pathogenesis and clinical features between early onset AD and late onset AD, they are known to be markedly similar in terms of their biological profiles including abnormalities in amyloid and tau biomarkers^31^. Despite inclusion of early onset AD patients, there was no difference in the proportion of patients who were homozygotes or heterozygotes for APOε4 allele, which may be due to small sample size in the AD group and selection bias. Fourth, previous study showed that there were significant differences in automated volumetric measurement across imaging sites which may be due to variability in scanner characteristics^50^ and reproducibility for automated volumetric analysis was not tested.

In conclusion, this study demonstrated the robust performance of automated brain volumetry on MRI and quantitative measurement of tau deposition on [18F] THK-5351 PET in discrimination of AD spectrum with added value of automated brain volumetry in discriminating AD from HC. The cingulate isthmus and inferior parietal lobule were identified as significant brain regions in discriminating AD on the AD spectrum, and may be used as adjunct to the traditionally used hippocampal volume.

## Supplementary Information

Supplementary Information.

## Abbreviations

AD: Alzheimer’s disease
HC: Healthy control
MCI: Mild cognitive impairment
AUROC: Area under the receiver operating characteristics
MAO-B: Monoamine oxidase B
SUVRs: Standard uptake value ratios
ANOVA: Analysis of variance
FDR: False discovery rate
