# Supplementary material for: Combination of automated brain volumetry on MRI and quantitative tau deposition on THK-5351 PET to support diagnosis of Alzheimer’s disease
Source: Sci Rep. 2021 May 14;11:10343. doi: 10.1038/s41598-021-89797-x (PMC8121780; doi:10.1038/s41598-021-89797-x)
Supplement: Supplementary file 1 — Supplementary Information. [file 41598_2021_89797_MOESM1_ESM.docx]

**Combination of automated brain volumetry on MRI and quantitative tau deposition on THK-5351 PET to support diagnosis of Alzheimer’s disease**

Minjae Kim^1^, Sang Joon Kim^1^, Ji Eun Park^1^, Jessica Yun^1^, Woo Hyun Shim^1^, Jungsu S. Oh^2^, Minyoung Oh^2^, Jee Hoon Roh^3^, Sang Won Seo^4^, Seung Jun Oh^2^ and Jae Seung Kim^2^

¹Department of Radiology and Research Institute of Radiology, Asan Medical Center, University of Ulsan College of Medicine, Seoul 05505, South Korea

^2^Department of Nuclear Medicine, Asan Medical Center, University of Ulsan College of Medicine, Seoul 05505, South Korea

³Department of Neurology, Asan Medical Center, University of Ulsan College of Medicine, Seoul 05505, South Korea

^4^Department of Neurology, Samsung Medical Center, Sungkyunkwan University School of Medicine, 81 Irwon-ro, Kangnam-ku, Seoul 06351, South Korea

Corresponding authors: (co-correspondence)

1) Sang Joon Kim, M.D., Ph.D.

Department of Radiology and Research Institute of Radiology, Asan Medical Center, University of Ulsan College of Medicine, 88 Olympic-ro 43-gil, Songpa-Gu, Seoul 05505, South Korea.

Phone: 82-2-3010-3953.

E-mail: sjkimjb5@gmail.com, sjkimjb@amc.seoul.kr

2) Jae Seung Kim, M.D., Ph.D.

Department of Nuclear Medicine, Asan Medical Center, University of Ulsan College of Medicine, 88 Olympic-ro 43-gil, Songpa-Gu, Seoul 05505, South Korea.

Phone: 82-2-3010-4594.

E-mail: jaeskim@amc.seoul.kr

**Supplementary Table 1.** Comparison of volumes using automated MR volumetry software and SUVRs of Braak ROIs in AD, MCI and HC

|  |  | **HC** | **MCI** | **AD** | **F-value** | **Degrees of freedom** | ***P*-value** | *Post-hoc analysis* | | |
| --- | --- | --- | --- | --- | --- | --- | --- | --- | --- | --- |
| **Volumes (mL)** |  |  |  |  |  |  |  | ***MCI vs. HC*** | ***AD vs. HC*** | ***AD vs. MCI*** |
| Hippocampus | | 7.41 | 6.36 | 5.43 | 12.674 | 2, 110 | 0.015 | 0.005 | < 0.001 | 0.037 |
| Entorhinal cortex | | 6.01 | 5.38 | 4.01 | 3.817 | 2, 110 | 0.001 | 0.221 | 0.001 | 0.001 |
| Superior temporal | | 28.16 | 27.12 | 22.44 | 1.794 | 2, 110 | 0.008 | 0.592 | < 0.001 | 0.001 |
| Middle temporal | | 25.83 | 25.23 | 20.18 | 5.583 | 2, 110 | 0.001 | 0.841 | < 0.001 | < .001 |
| Basal ganglia | | 18.70 | 18.04 | 16.11 | 2.164 | 2, 110 | 0.011 | 0.035 | < 0.001 | 0.006 |
| Amygdala | | 3.23 | 2.88 | 2.39 | 7.983 | 2, 110 | 0.011 | 0.012 | < 0.001 | 0.003 |
| Anterior cingulate | | 6.09 | 5.98 | 5.05 | 6.014 | 2, 110 | 0.002 | 0.842 | 0.006 | 0.013 |
| Posterior cingulate | | 3.63 | 3.68 | 3.13 | 7.677 | 2, 110 | < 0.001 | 0.953 | 0.041 | 0.011 |
| Cingulate isthmus | | 4.77 | 4.59 | 3.85 | 10.083 | 2, 110 | < 0.001 | 0.622 | 0.001 | 0.006 |
| Anterior middle frontal | | 15.35 | 14.67 | 12.38 | 3.461 | 2, 110 | 0.004 | 0.161 | < 0.001 | 0.007 |
| Inferior parietal lobule | | 27.31 | 25.83 | 21.82 | 18.072 | 2, 110 | < 0.001 | 0.362 | < 0.001 | 0.005 |
| Supramarginal | | 18.03 | 16.37 | 14.02 | 12.976 | 2, 110 | < 0.001 | 0.072 | < 0.001 | 0.021 |
| **SUVR (ratio)** | |  |  |  |  |  |  |  |  |  |
| Braak I/II | | 1.89 | 2.04 | 2.02 | 3.622 | 2, 110 | 0.011 | 0.031 | 0.191 | 1.00 |
| Braak III/IV | | 1.46 | 1.58 | 1.65 | 10.214 | 2, 110 | < 0.001 | 0.004 | < 0.001 | 0.231 |
| Braak V/VI | | 1.20 | 1.29 | 1.38 | 19.125 | 2, 110 | < 0.001 | 0.002 | < 0.001 | 0.002 |

Degrees of freedom are presented as within-group followed by between-group degrees of freedom. *P*-values for volumes were corrected using false discovery rate. P-values for SUVR were Bonferroni-corrected.

**Supplementary Table 2.** Univariate and multivariate logistic regression analysis of SUVRs of brain regions for discriminating AD from HC and MCI

|  | AD vs. HC | | | | AD vs. MCI | | | |
| --- | --- | --- | --- | --- | --- | --- | --- | --- |
| Variable | Univariate analysis | | Multivariate analysis | | Univariate analysis | | Multivariate analysis | |
|  | β coefficient (95% CI) | *P value* | β coefficient (95% CI) | *P value* | β coefficient (95% CI) | *P value* | β coefficient (95% CI) | *P value* |
| Pars opercularis | 10.75 (5.12, 16.39) | **< 0.001** |  |  | 4.64 (1.21, 8.08) | **0.008** |  |  |
| Pars orbitalis | 8.49 (3.71, 13.26) | **< 0.001** |  |  |  |  |  |  |
| Pars triangularis | 9.34 (4.30, 14.42) | **< 0.001** |  |  | 3.94 (0.25, 7.64) | **0.037** |  |  |
| Caudal middle frontal | 10.02 (5.00, 15.05) | **< 0.001** |  |  | 4.85 (1.98, 7.72) | **0.001** |  |  |
| Lateral orbitofrontal | 5.81 (1.95, 9.67) | **0.003** |  |  |  |  |  |  |
| Rostral middle frontal | 9.32 (4.46, 14.19) | **< 0.001** |  |  | 4.28 (1.66, 6.91) | **0.001** |  |  |
| Superior frontal | 8.01 (3.74, 12.28) | **<0.001** |  |  | 3.45 (0.40, 6.51) | **0.027** |  |  |
| Frontal pole | 4.61 (0.86, 8.36) | **0.016** |  |  |  |  |  |  |
| Entorhinal | 2.38 (0.08, 4.69) | **0.043** |  |  |  |  |  |  |
| Fusiform | 5.35 (1.98, 8.71) | **0.002** |  |  |  |  |  |  |
| Lingual | 10.63 (4.28, 16.97) | **0.001** |  |  | 4.74 (0.87, 8.60) | **0.016** |  |  |
| Inferior temporal | 5.12 (2.18, 8.06) | **0.001** |  |  |  |  |  |  |
| Middle temporal | 6.14 (2.77, 9.51) | **< 0.001** |  |  | 2.55 (0.45, 4.66) | **0.017** |  |  |
| Superior temporal | 7.55 (2.98, 12.11) | **0.001** | -10.82 (-22.34, 0.70) | 0.066 |  |  |  |  |
| Precuneus | 7.35 (3.80, 10.90) | **< 0.001** |  |  | 5.33 (2.52, 8.14) | **< 0.001** |  |  |
| Supramarginal | 10.59 (5.57, 15.62) | **< 0.001** | 18.89 (7.52, 30.25) | **0.001** | 7.45 (3.80, 11.10) | **< 0.001** | 10.16 (5.50, 14.82) | **< 0.001** |
| Inferior parietal | 7.55 (3.74, 11.36) | **< 0.001** |  |  | 4.03 (1.96, 6.09) | **< 0.001** |  |  |
| Superior parietal | 9.85 (4.83, 14.86) | **< 0.001** |  |  | 5.87 (2.77, 8.96) | **< 0.001** |  |  |
| Cuneus | 8.84 (3.57, 14.11) | **0.001** |  |  | 3.40 (0.06, 6.73) | **0.046** |  |  |
| Pericalcarine | 7.81 (2.68, 12.94) | **0.003** |  |  |  |  |  |  |
| Lateral occipital | 7.31 (2.89, 11.72) | **0.001** |  |  | 2.61 (0.52, 4.70) | **0.014** |  |  |
| Paracentral | 9.90 (4.36, 15.43) | **< 0.001** |  |  | 4.41 (0.82, 8.00) | **0.016** |  |  |
| Postcentral | 11.90 (5.03, 18.76) | **0.001** |  |  | 5.46 (0.83, 10.09) | **0.021** |  |  |
| Precentral | 11.38 (4.75, 18.00) | **0.001** |  |  |  |  |  |  |
| Cingulate isthmus | 7.73 (3.80, 11.66) | **< 0.001** |  |  | 4.90 (1.93, 7.87) | **0.001** |  |  |
| Posterior cingulate | 6.52 (3.08, 9.97) | **< 0.001** |  |  | 4.14 (1.42, 6.85) | **0.003** |  |  |
| Rostral anterior cingulate | 3.08 (0.08, 6.09) | **0.044** |  |  |  |  |  |  |
| Insula | 5.63 (1.71, 9.56) | **0.005** |  |  |  |  |  |  |

**Supplementary Table 3.** Performance using SUVRs of selected brain regions and volumes of Braak ROIs in discriminating AD from HC and MCI

| **AD vs. HC** | **AUROC** | **Sensitivity (%)** | **Specificity (%)** | **Accuracy (%)** |
| --- | --- | --- | --- | --- |
| SUVR of selected brain region (supramarginal) | 0.90 (0.82, 0.98) | 88.8 | 87.5 | 84.5 |
| Volumes of Braak ROIs (Braak I/II, III/IV, V/VI) | 0.81 (0.70, 0.92) | 61.5 | 90.6 | 77.6 |
| **AD vs. MCI** | **AUROC** | **Sensitivity (%)** | **Specificity (%)** | **Accuracy (%)** |
| SUVRs of selected brain regions (supramarginal) | 0.79 (0.66, 0.92) | 73.1 | 85.5 | 81.5 |
| Volumes of Braak ROIs (Braak I/II, III/IV, V/VI) | 0.60 (0.47, 0.73) | 53.8 | 67.3 | 63.0 |

Note: Numbers in parentheses are 95% confidence intervals. AUROC = area under the receiver operating characteristics curve.
